# Lyme Disease Agent Reservoirs *Peromyscus leucopus* and *P. maniculatus* Have Natively Inactivated Genes for the High-Affinity Immunoglobulin Gamma Fc Receptor I (CD64)

**DOI:** 10.3390/pathogens12081056

**Published:** 2023-08-18

**Authors:** Alan G. Barbour, Jonathan V. Duong, Anthony D. Long

**Affiliations:** 1 Department of Microbiology & Molecular Genetics, School of Medicine, University of California Irvine, Irvine, CA 92697, USA; jvduong@uci.edu; 2 Department of Medicine, School of Medicine, University of California Irvine, Irvine, CA 92697, USA; 3 Department of Ecology & Evolutionary Biology, School of Biological Sciences, University of California Irvine, Irvine, CA 92697, USA; adlong@uci.edu

**Keywords:** tick-borne disease, Cricetidae, retroelement, zoonosis, Fc receptor, deer mouse

## Abstract

The abundant and widely distributed deermice *Peromyscus leucopus* and *P. maniculatus* are important reservoirs for several different zoonotic agents in North America. For the pathogens they persistently harbor, these species are also examples of the phenomenon of infection tolerance. In the present study a prior observation of absent expression of the high-affinity Fc immunoglobulin gamma receptor I (FcγRI), or CD64, in *P. leucopus* was confirmed in an experimental infection with *Borreliella burgdorferi*, a Lyme disease agent. We demonstrate that the null phenotype is attributable to a long-standing inactivation of the Fcgr1 gene in both species by a deletion of the promoter and coding sequence for the signal peptide for FcγRI. The Fcgr1 pseudogene was also documented in the related species *P. polionotus*. Six other *Peromyscus* species, including *P. californicus*, have coding sequences for a full-length FcγRI, including a consensus signal peptide. An inference from reported phenotypes for null Fcgr1 mutations engineered in *Mus musculus* is that one consequence of pseudogenization of Fcgr1 is comparatively less inflammation during infection than in animals, including humans, with undisrupted, fully active genes.

## 1. Introduction

A common feature of vertebrate reservoirs for zoonotic agents is that they are little disabled by the otherwise pathogenic microorganisms they host. The infection may be persistent, but it is restrained. If there is inflammation, it minimally affects fitness. These are characteristics of infection tolerance, which broadly includes both plants and animals [1]. While pathogenetic mechanisms of microbes and the pathology they cause in the host remain a priority for infectious diseases research, there is increasing attention on mitigating mechanisms that maintain the host’s health during an active infection. In addition to host defences that limit the microbe in its harm, infection tolerance mechanisms also include moderation or reversal of immune system activities that are maladaptive in the extent of their collateral damage. 

A group of animals that are models for reservoir competence and infection tolerance is the North American rodent genus *Peromyscus*, also known as deermice [2]. Two deermouse species of public health importance as reservoirs are *Peromyscus leucopus*, the white-footed deermouse, and *P. maniculatus*, the North American deermouse. The geographic distributions of these two abundant species overlap in the longitudinal midsection of the continent, with the range of *P. leucopus* exclusively extending to the Atlantic coast and that of the *P. maniculatus* group reaching the Pacific coast [3,4]. *P. leucopus* is a reservoir for the agents of several diseases transmitted to humans and domestic animals by *Ixodes* species ticks. These diseases include Lyme disease (LD), babesiosis, anaplasmosis, hard tick-borne relapsing fever or *Borrelia miyamotoi* disease, Powassan virus encephalitis, and a form of ehrlichiosis. *P. maniculatus* likewise serves as a reservoir for the LD agent *Borreliella (Borrelia) burgdorferi* in parts of its range [5,6], but it is better known as a reservoir for the Sin Nombre hantavirus [7]. 

While *P. leucopus* and *P. maniculatus* are mouse-like in appearance and size, they are in the family Cricetidae, together with hamsters, voles, and woodrats, and not the family Muridae, which includes “Old World” mice and rats, such as *Mus* and *Rattus* [8]. In the family Cricetidae, the closest identified outgroup to the genus *Peromyscus* among rodents with sequenced genomes is *Onychomys torridus*, the southern grasshopper mouse [9,10].

Our long-term project is a characterization of the strategies that *P. leucopus* deploys to thrive in its environment, all the while infected with one or more microbes that cause disease in humans and laboratory mice. In pursuit of this goal, we compared *P. leucopus* and *M. musculus* for their transcriptional responses in the blood, spleen, and liver to the endotoxin lipopolysaccharide (LPS) from the Gram-negative bacterium *Escherichia coli* [11,12]. There were hundreds of differentially expressed genes (DEG) between LPS-treated and control animals. Among the DEGs distinguishing deermice and mice in their responses to LPS was Fcgr1, the gene for high-affinity immunoglobulin gamma Fc receptor I (FcγRI), also known as CD64 [11]. Fcgr1 increased in transcription in the blood, spleen, and liver of *M. musculus* after LPS injection. In contrast, there were few, if any, transcripts corresponding to the locus annotated as Fcgr1 in *P. leucopus* under either condition in blood or either organ. FcγRI has plausible relevance for infection tolerance phenomenon; mice with knockouts of this gene display less inflammation than wildtype mice in different disease models [13,14]. In addition, there may be a non-canonical relationship between this protein and *B. burgdorferi*; Carreras-González et al. reported a direct interaction between FcγRI on mouse macrophages and *B. burgdorferi* in vitro without the requirement of opsonizing antibody [15]. 

In the present study we analyzed the results of a replicate LPS experiment with outbred *M. musculus* replacing inbred BALB/c mice for comparison with outbred *P. leucopus* transcription in the blood [12]. We also assessed the transcription of Fcgr1 and other Fc receptors in the spleens of *P. leucopus* either infected with *B. burgdorferi* or not. We confirmed the original observation and further defined the Fcgr1 gene locus in *P. leucopus*. Finally, we characterized the locus in populations other than the colony that was the source for the *P. leucopus* genome sequence and extended the analysis of the Fcgr1 locus to other *Peromyscus* species. 

## 2. Materials and Methods

**Figure 1:**
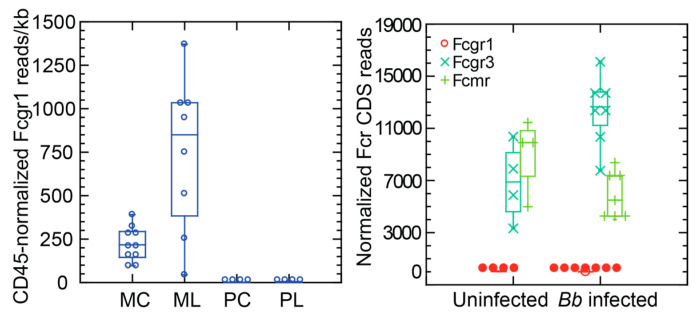
Transcription of Fcgr1 in *M. musculus* (M) and *P. leucopus* (P) under conditions of lipopolysaccharide (LPS) exposure (**left**) or infection of *P. leucopus* with *B. burgdorferi* (**right**). Left panel, the animals received either 10 µg LPS per g body mass (L) or were untreated controls (C); RNA extract was of whole blood obtained 4 h after injection. Right panel, the three Fc receptor (Fcr) targets were Fcgr1 and the coding sequences for low-affinity FcγRIII (Fcgr3; CD16) and the Fc fragment of IgM receptor (Fcmr). The RNA extract was of the spleen and was obtained 21 days after initiation of infection. Normalization of reads per kilobase for target transcripts was conducted with Ptprc, the gene for CD45, for blood samples (**left**) and by adjustment for total reads for a sample for the spleen samples (**right**).

**Animals.** *Peromyscus leucopus*, here also referred to as “deermice”, were of the outbred LL stock, which originated with 38 animals captured in North Carolina in the mid-1980’s and thereafter constituted a closed colony without sib–sib matings at the Peromyscus Genetic Stock Center at the University of South Carolina [16,17]. The LL stock animals for this study were bred and raised at the vivarium of University of California Irvine, an AAALAC approved facility. Severe combined immunodeficiency (SCID) *M. musculus* strain C.B.17 SCID mice were obtained from Charles River Laboratories. The study in which CD-1 *M. musculus* and LL stock *P. leucopus* were used in an experiment with lipopolysaccharide treatment is described in Milovic et al. [12]. That study was the source of sequence reads for the analysis of Figure 1. The source of the DNA extracts from blood samples from field-captured *P. leucopus* is given below.

**Infection model.** Adult *P. leucopus* of both sexes were infected on day 0 by subcutaneous injection of 10^4^ *B. burgdorferi* strain RST2-1, which had been provided by Klemen Strle of the Wadsworth Laboratory of the New York State Department of Health. The OspC genotype was K. The inoculum was a first passage culture in BSK-H medium (Sigma Aldrich, St. Louis, MO, USA) of spirochetes that had been propagated in the blood of SCID mice. On day 21 the *P. leucopus* were euthanized and ear skin, bladder, and spleen tissue were collected. DNA was extracted from the skin and bladder, and RNA was extracted from the spleen. The probe-based quantitative PCR assay for *B. burgdorferi* was carried out on DNA from the skin and bladder as described [18]. 

**Nucleic acid extractions.** The DNA of skin and bladder tissues was extracted and purified using the Qiagen DNeasy Blood and Tissue Kit according to the manufacturer’s instructions. Skin samples were treated with 30 µL of proteinase K at 20 mg/mL and 170 µL of Qiagen Buffer ATL tissue lysis reagent. This was followed by 12 h incubation at 56 °C on a rotating shaker before the final steps of the extraction kit. DNA samples were stored in elution Buffer AE (Qiagen, Valencia, CA, USA) at −20 °C. RNA was extracted from the spleen, which had been stored at −80 °C, using the RNeasy Mini Kit (Qiagen, Valencia, CA, USA) and homogenization with a stainless-steel bead on a TissueLyser (Qiagen, Valencia, CA, USA). Concentrations and purity of DNA extracts were measured using a Nanodrop ND-1000 spectrophotometer and a Qubit 2.0 (Invitrogen, Waltham, MA, USA) fluorometer. RNA integrity was further analyzed on an Agilent 2100 Bioanalyzer (Agilent Technologies, Santa Clara, CA, USA) with the Nano RNA chip. 

**Confirmation of genome assembly.** A 9954 bp fragment of the annotated Fcgr1 gene was amplified using the following forward and reverse primers: 5′-ATCCTAACGCCTGGATTGGGA and 5′-TGTCGGGATGTCAAGCGGCT. These encompassed a span from 227 nt 5′ of annotated start codon for Fcgr1 (positions 70,734,123–70,733,897 of chromosome 6; NC_051068) to 228 nt 3′ of annotated stop codon for Fcgr1 (positions 70,724,018–70,723,791). A LongAmp Taq PCR Kit (New England Bio Labs, Ipswich, MA, USA) was used, and the thermocycling conditions in a Bio-Rad T100 thermal cycler were as follows: 3 min at 95 °C, 40 cycles of 95 °C for 15 s, 66 °C for 9.1 m, and one 10 min final extension at 66 °C. A fragment of 1637 bp overlapping with and further upstream of the designated Fcgr1 gene was amplified using forward primer 5′-GTGACCCCTGTGAGCATTGT and reverse primer 5′-ACCAGAGCAAACTGCCTTGA. These were positions 70,735,686–70,735,705 and 70,734,088–70,734,069 of chromosome 6, respectively. PCR master mix comprised Taq DNA Polymerase with 10× ThermoPol Reaction Buffer set (New England Biolabs Inc.), dNTP/dUTP Mix (Thermo Fisher Scientific, Waltham, MA, USA), 50 mM magnesium chloride (Invitrogen, Waltham, MA, USA), and the addition of uracil-DNA-glycosylase (UDG) (Thermo Fisher Scientific, Waltham, MA, USA). Final concentrations of 0.25 units of UDG, 200 nM of each primer, 2.5 units of Taq polymerase, 0.2 mM dNTP, 0.4 mM dUTP, 3 mM magnesium chloride, and 10× ThermoPol Reaction Buffer diluted to 1× concentration with sufficient nuclease free water were used in each 25 μL PCR reaction. The thermocycling conditions were 37 °C for 10 min, 95 °C for 10 min, followed by 40 cycles of 95 °C for 15 s, 64 °C for 30 s, and 72. The two amplified fragments were subjected to Sanger dideoxy sequencing at Azenta Life Sciences (San Diego, CA, USA).

**Fcgr1 genotyping.** A 392 fragment that contained the stop codon in the reading frame of the Fcgr1 gene of *P. leucopus* was amplified with forward primer 5′-TGAGATCTGGCCTCTTGGACT and reverse primer 5’-CCGTTGTAAGTCAGGTGAGGA. These corresponded to positions 70,726,812–70,726,792 and 70,726,441–70,726,421 of chromosome 6, respectively. The Taq DNA Polymerase and master mix were those described above. PCR conditions on a Bio-Rad T100 Thermal Cycler (Bio-Rad Laboratories, Hercules, CA, USA) were as follows: 37 °C for 10 min, 95 °C for 10 min, followed by 40 cycles of 95 °C for 10 s, 63.5 °C for 30 s, and 72 °C for 45 s. The PCR amplicon was isolated from an agarose gel containing SYBR Safe DNA Gel Stain and purified using the NucleoSpin Gel and a PCR Clean-Up Kit (Takara, Kusatsu City, Shiga Prefecture, Japan). Sanger dideoxy sequencing with custom primers was performed on purified PCR products at Azenta Life Sciences. Trace files were visualized with FinchTV (https://digitalworldbiology.com/FinchTV, accessed on 11 February 2023) to distinguish homozygotes from heterozygotes at the position of interest.

**RNA-seq.** The RNA-seq procedure for the blood samples of *P. leucopus* and *M. musculus* treated with lipopolysaccharide or saline control have been described [12]. RNA extracts of the spleen for production of cDNA libraries were prepared with the Illumina TruSeq Stranded mRNA kit utilizing a ribosomal RNA depletion step. After normalization and multiplexing, the libraries were sequenced at the University of California Irvine’s Genomics Research and Technology Hub on an Illumina NovaSeq 6000 instrument with paired-end chemistry and 150 cycles to achieve ~60–80 million reads per sample. The quality of sequencing reads was analyzed using FastQC (Babraham Bioinformatics). The reads were trimmed of low-quality reads (Phred score of <15) and adapter sequences and corrected for poor-quality bases using Trimmomatic (http://www.usadellab.org, accessed on 26 July 2023). RNA-seq of the selected set of protein coding sequences (CDS) was carried out using CLC Genomics Workbench v. 23 (Qiagen). Paired-end reads were mapped with a length fraction of 0.35 for ~150 nt reads, a similarity fraction of 0.9, and penalties of 3 for mismatch, insertion, or deletion. For blood samples to adjust for differences in the numbers of white cells, expression values for individual samples were unique reads for a given target transcript normalized for the number of unique reads mapped to the coding sequence of Ptprc, which encodes CD45, a marker for both granulocytes and mononuclear cells in the blood, for that sample [12]. For the RNA-seq of spleen extracts, the unique reads for a given target were normalized across all samples for total reads for that sample and expressed as reads per kilobase. 

**Additional sequence analysis.** Dot matrix plots of two aligned sequences were produced with MAFFT v. 7 with a threshold setting of 39 (https://mafft.cbrc.jp, accessed on 6 August 2022). A RepeatMasker v. 4.1 (http://repeatmasker.org/cgi-bin/WEBRepeatMasker, accessed on 21 August 2022) and SINEBase (https://sines.eimb.ru, accessed on 22 August 2022) [19] were used to identify SINE and other retroelements. For signal peptide prediction, a SignalIP v. 6.0 (https://services.healthtech.dtu.dk/services/SignalP-6.0/, accessed on 4 August 2022) was used [20]. A SeaView v. 4 was used for alignments and for generation of PhyML-based maximum likelihood phylogenetic trees with a GTR model of evolution, 4 rate classes, and evaluation of nodal support by bootstrap with 1000 iterations [21].

**Reference sequences.** The FcγRI protein sequences (GenBank accession) were of *Cricetulus griseus* (XP_007628905.3), *Gorilla gorilla* (XP_030865403.1), *Hipposideros armiger* (XP_019490029.1), *Homo sapiens* (NP_001365735.1), *Mesocricetus auratus* (XP_012979241.1), *Microtus ochrogaster* (XP_013205295.1), *Microtus oregoni* (XP_041494111.1), *Mus musculus* (NP_034316.1), *Mus pahari* (XP_021051658.1), *Onychomys torridus* (XP_036046441.1), *Peromyscus leucopus* (XP_037063200.1), *Peromyscus maniculatus bairdii* (XP_015856558.2), *Rattus norvegicus* (NP_001094306.1), *Rattus rattus* (XP_032753583.1), *Rhinolophus ferrumequinum* (XP_032949698.1), and *Rhinolophus sinicus* (XP_019595422.1). Alignment of amino acids based on positions 31–193 of the *M. musculus* reference sequence is shown in Figure S1; this corresponds to positions 33–194 with one gap of the *P. leucopus* sequence. This region comprises the first and second immunoglobulin-like domains.

The Whole Genome Shotgun sequence files or chromosome-scale assemblies were for *P. attwateri* (CABHPP010146044), *P. aztecus* (CABHPQ010140196), *P. californicus insignis* (GCA_007827085.3), *P. eremicus* (CACRXM010000006), *P. leucopus* (GCF_004664715.2), *P. maniculatus bairdii* (GCF_003704035.1), *P. maniculatus sonoriensis* (JAOPKX010000000), *P. melanophrys* (CABHPR010082406), and *P. polionotus* (RCWS02265928).

Mitochondrial cytochrome-*b* nucleotide sequences were for *P. attwateri* (NC_035593), *P. aztecus* (NC_035596), *P. boylii* (MZ433362), *P. californicus insignis* IS (OP524493), *P. eremicus* (AY322503), *P. leucopus* LL (MG674647), *P. maniculatus bairdii* BW (MH260579), *P. maniculatus sonoriensis* SM (EU140796), *P. melanophrys* (NC_035611), *P. polionotus* (NC_035571), and the southern grasshopper mouse *Onychomys torridus* (KY754082).

The annotated Fcgr1 gene regions for *P. leucopus* LL stock isolate 20307 and *P. californicus insignis* IS are GenBank accession numbers OP292976 and OP573260, respectively. The sequence of the mRNA for the FcγRI of *P. boylii* was OP524494.

## 3. Results

### 3.1. Fcgr1 Locus Transcription

We used RNA-seq data for blood collected from 20 outbred *P. leucopus* and 20 outbred *M. musculus*, equally divided by sex, in an experiment with blood samples obtained 4 h after injection of either *Escherichia coli* LPS at a dose of 10 µg/g or sterile saline alone [12]. For the matching reference sets for each rodent species we used the mRNA coding sequences for Fcgr1 beginning after the signal peptides. As a reference transcript for the targeted RNA-seq to normalize across samples for differences in white cell concentrations in the blood, we used Ptprc, the gene for protein tyrosine phosphatase receptor type C, also known as CD45 and commonly used as a marker for all white cells in flow cytometry [22].

The left panel of Figure 1 shows that there was baseline transcription of the Fcgr1 coding sequence in the blood of control outbred mice and an increase in most of the mice after exposure to LPS. In contrast, there was little or no detectable transcription of the orthologous sequence of *P. leucopus* either in the presence or absence of LPS. 

For an assessment of Fcgr1 locus transcription under conditions of relevance for *P. leucopus’* role as a reservoir, we infected seven adult *P. leucopus* (three females and four males) with *B. burgdorferi* by subcutaneous injection and collected blood and tissue samples 3 weeks later. By quantitative PCR of ear skin and bladder biopsies, all injected animals were infected. These animals were compared with four uninfected controls (two females and two males) by targeted RNA-seq. For this analysis the comparisons with Fcgr1 were Fcgr3 and the protein Fc fragment of IgM receptor (Fcmr), another low affinity Fc receptor. As was noted for the blood of *P. peromyscus*, there was scant or no detectable transcription of the Fcgr1 coding sequence under either condition. There was appreciable transcription of both Fcgr3 and Fcmr in the control group. There was an increase in Fcgr3 with infection, but lower transcription of Fcmr. 

The present study’s findings together with our previous observations led us to conclude that expression of a Fcgr1 ortholog was not occurring in *P. leucopus* blood or spleen, two locations where it would be expected. Accordingly, we next examined this locus in more depth for evidence of inactivation or other explanation for transcriptional silence.

### 3.2. Fcgr1 Locus of P. leucopus

A 10,074 bp sequence (LOC114682367) spanning positions 70,724,019 to 70,734,092 of the minus strand of *P. leucopus* chromosome 6 (NC_051068) was annotated as the gene for a FcγRI-like protein of *P. leucopus*. This corresponded to a Fcgr1-like gene (LOC102922896) of the *P. maniculatus* genome 10,087 bp in length. The *P. leucopus* genome was the product of hybrid assemblies of long reads and short reads [23], so it was possible that there were errors in the assembly. Accordingly, we carried out PCR amplification of the entire locus followed by Sanger sequencing for *P. leucopus*. The sequence of the index animal for the genome was confirmed by this independent method. 

**Figure 2:**
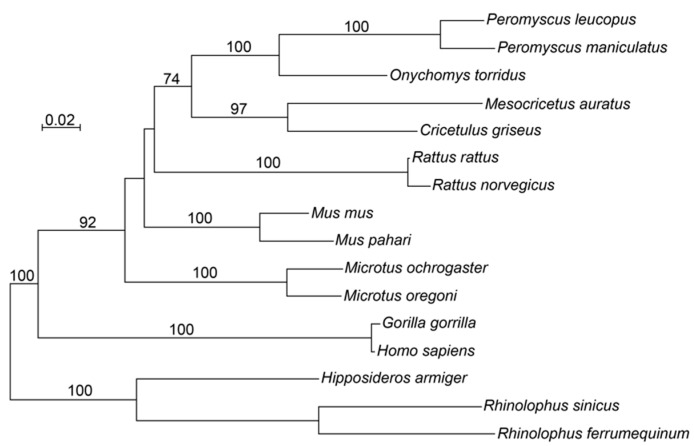
Distance phylogram of aligned amino acid sequences for the first two immunoglobulin-like domains of FcγRI of *Peromyscus leucopus* and *P. maniculatus* and selected other rodents, primates, and bats. The alignments are shown in Figure S1, and the sources of sequences are given in the Methods. The method for evolutionary distance was the Poisson Distance Correction, and the tree building algorithm was BioNJ. The numbers along branches indicate % bootstrap support out of 100 iterations. The length marker is the distance value.

Four exons, arrayed in the order of 539, 261, 696, and 77 bp in length, were identified in the predicted mRNA of 1573 nt of XM_037207305.1. These exons encode a predicted protein of 458 amino acids (XP_037063200). Using this protein sequence for a blastp search (https://blast.ncbi.nlm.nih.gov, accessed on 4 August 2022), we found that the only proteins in GenBank with 55% or greater identity for over 55% of their lengths were FcγRI proteins of other rodents, including the golden hamster *Mesocricetus auratus*, *M. musculus*, and *Rattus norvegicus*. Alignment of the deduced protein sequence of *P. leucopus* with selected other rodents, as well as two primates and three Microchiroptera bats, showed that beginning with residue 33 of the *P. leucopus* sequence, it was homologous, with identities of >72% and few if any gaps, to corresponding fragments of FcγRI proteins of other rodents (Figure S1). The phylogram of Figure 2 of the aligned sequences after position 32 shows *P. leucopus* in a cluster with *P. maniculatus* and the cricetines *M. auratus*, the Chinese hamster *Cricetulus griseus*, and the southern grasshopper mouse *Onychomys torridus*, a topology that recapitulates other phylogenies for rodents [24]. 

**Figure 3:**
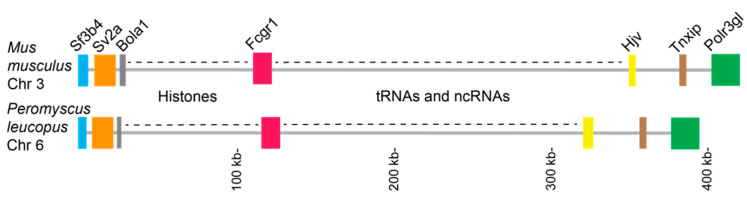
Schematic physical map of Fcgr1 gene locus regions of chromosome (Chr) 3 of *M. musculus* and chromosome 6 of *P. leucopus* with the locus for Fcgr1 (red). Other genes, their approximate sizes, and their positions are indicated. Regions rich in histones and histone-type genes or in tRNAs and non-coding RNAs (ncRNAs) in both animals are denoted. The ruler of lengths is at the bottom.

Further evidence that this locus is orthologous to the Fcgr1 genes of other rodents was its location in the *P. leucopus* genome. In this species, as well as in *P. maniculatus* [25], it is on chromosome 6 [23]. In *M. musculus* Fcgr1 is on chromosome 3, but this chromosome is largely syntenic over its length with chromosome 6 of *Peromyscus* [23]. In both *P. leucopus* and *M. musculus* the Fcgr1 gene has arrays of tRNAs and ncRNAs on its right flank and histone genes on its left flank in their respective chromosomes (Figure 3). The genes beyond these arrays on each side are the same in identity, order, and approximate distance in both species. From these studies we concluded that the assembly in this region is correct. The locus of *P. leucopus*, a member of the family Cricetidae, shares its descent with the Fcgr1 gene of a representative of the family Muridae. 

### 3.3. Deduced Protein of Fcgr1 of Peromyscus

**Figure 4:**
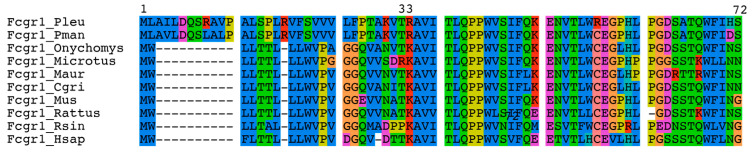

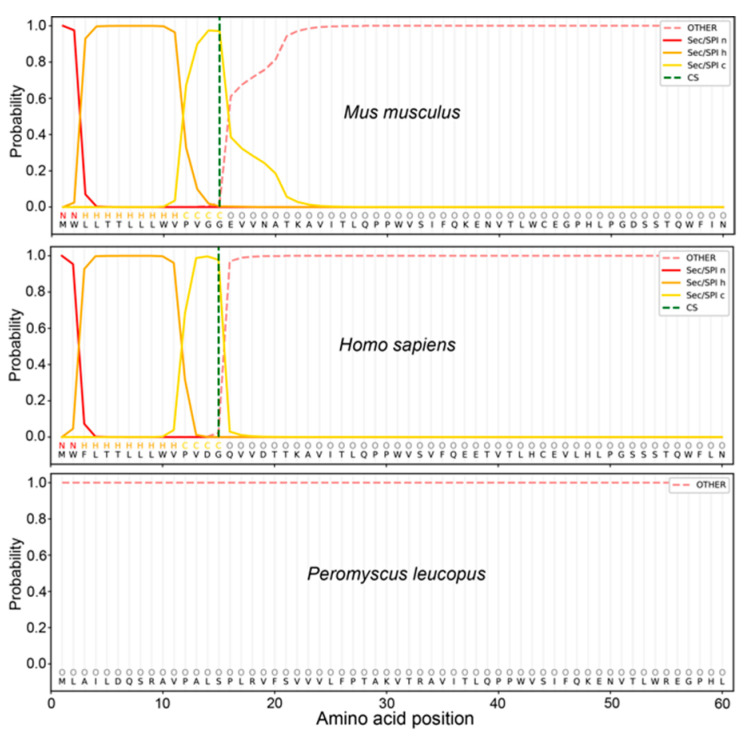
N-terminal sequences of FcγRI and FcγRI-like proteins of *P. leucopus*, *P. maniculatus*, and selected other rodents and primates. Upper panel, alignment of first 72 amino acids of the predicted open reading frame of *P. leucopus* FcγRI-like protein and corresponding regions of other species. Represented are *P. leucopus* (Pleu), *P. maniculatus* (Pman), *Onychomys torridus* (Onycho), *Microtus ochrogaster*, *Me. auratus* (Maur) *C. griseus* (Cgri), *M. musculus* (Mus), *R. norvegicus* (Rattus), *Rhinolophus sinicius* (Rsin), and *Homo sapiens* (Hsap). Lower panel, predictions of signal peptides by the SignalIP v. 6 algorithm for the first 60 amino acids of the proteins of *M. musculus*, *H. sapiens*, and *P. leucopus*. The amino acids are shown at the bottom of each graph. The x-axis is the calculated probability. The likelihoods of N-terminal (n), hydrophobic (h), and C-terminal regions of a Sec-Signal Peptidase 1-type signal peptide are indicated by red, orange, and yellow lines, respectively. The position at the vertical dashed line is the predicted cleavage site (CS).

We could not completely exclude expression of the predicted coding sequence under some condition not examined here. If this could occur, might the expressed protein be functional? The upper panel of Figure 4 is an alignment of the N-terminal ends of the deduced protein sequences for FcγRI or FcγRI-like proteins of *P. leucopus*, *P. maniculatus*, *O. torridus*, the prairie vole *Microtus ochrogaster*, *Me. auratus*, *C. griseus*, *R. norvegicus*, *M. musculus*, the Chinese rufous horseshoe bat *Rhinolophus sinicus*, and *Homo sapiens*. From amino acids 1 through 32, the predicted proteins of *P. leucopus* and *P. maniculatus* are unlike the proteins of the other mammals in the set. What serves as the signal peptide for the other proteins would be replaced in these two *Peromyscus* proteins by an N-terminal sequence that is not predicted to function as a signal peptide (Figure 4 lower panel). This stands in contrast to the N-terminal amino acid sequences of the *M. musculus* and *H. sapiens* proteins, which were accurately predicted as signal peptides. Therefore, even if the putative coding sequence was transcribed and translated, we would not expect that product to be transported and then positioned in the membrane to function as a receptor. 

### 3.4. The 5′ End and Flank of Peromyscus Fcgr1

**Figure 5:**
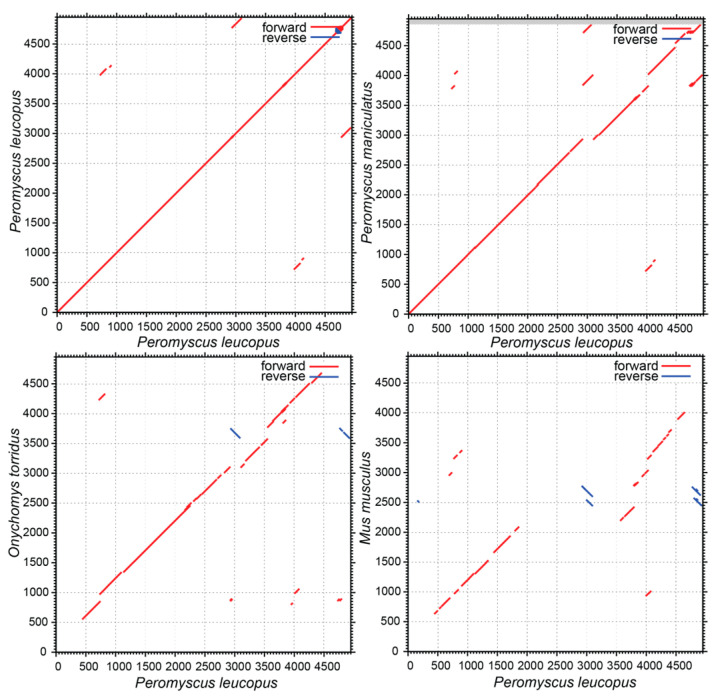
Dot matrix plots of the first 5000 nucleotides of the predicted Fcgr1 gene loci of *P. leucopus* (**upper left**) *P. maniculatus* (**upper right**), *O. torridus* (**lower left**) and *M. musculus* (**lower right**) against the aligned *P. leucopus* sequence in each panel. The threshold for the plots by the MAFFT algorithm was a score of 39. Direct repeats are indicated by red lines parallel to the diagonal, and inverted repeats are indicated by blue lines perpendicular to the diagonal.

The promoter for Fcgr1 of mouse and human is within ~190 nt of the start of [26]. We added this flanking mouse sequence to the annotated Fcgr1 gene of *M. musculus* and, correspondingly, added approximately the same lengths flanking sequence to the annotated Fcgr1 genes of *P. leucopus*, *P. maniculatus*, and *O. torridus*, the cricetine most closely related to *Peromyscus* among genome sequences. When these sequences were aligned over the first 5000 positions and pairwise dot matrix plotting carried out, the synteny and high similarity of *P. leucopus* and *P. maniculatus* were apparent (Figure 5). There was also evidence of homology between the *P. leucopus, O. torridus*, and *M. musculus* sequences over much of their lengths, albeit with some disjunctions. However, there was no evident similarity between the first ~500 nt for *P. leucopus* and either *O. torridus* or *M. musculus*. This corresponds to the region containing promoter and exon 1 for the *M. musculus* gene. What was annotated as “exon 1” with the translational start of *P. leucopus*’ Fcgr1-like gene does not occur until just before exon 2 of *M. musculus* and *O. torridus* in the alignment. 

Since it was possible that the rearrangement or deletion in *P. leucopus* was limited to the LL stock population, a breeding colony which had been closed since the 1980’s, we determined the sequences of the corresponding region of an animal drawn from the inbred strain GS16A1 population of *P. leucopus*, which was started in the 1960’s from animals captured in Illinois, an animal of an outbred closed colony derived from wild population of New York, and a wild animal from a population in Connecticut [27]. The sequences were 96–97% identical to that for the LL stock reference. Similarly, the corresponding sequence on scaffold JAOPKW010000002 of a *P. maniculatus sonoriensis* was 98% identical to that of the reference genomes of *P. maniculatus bairdii* and *P. polionotus*, the oldfield deermouse, and closely related to *P. maniculatus* [24]. 

Another *Peromyscus* species for which there was a near-complete genome assembly is the California deermouse *P. californicus*. This animal is predicted to have a FcγRI protein (GenBank accession XP_052586848.1) with a signal peptide sequence similar to that of the mouse protein. In *P. aztecus*, the Aztec mouse, a FcγRI protein was noted in the genome annotation, but the predicted protein lacked the N-terminal and C-terminal regions of other FcγRI proteins. Examination of whole genome shotgun contig CABHPQ010140196 containing this coding sequence, revealed sequences orthologous for exon 1 (positions 25,456–25,511) and exon 2 (25,889–258,909) of the *M. musculus* gene. These exons would encode the signal peptide for a FcγRI. We similarly identified encoding sequences for a signal peptide in deposited whole genome contigs for the Texas deermouse *P. attwateri*, cactus deermouse *P. eremicus*, and plateau deermouse *P. melanophrys*. Transcripts encoding FcgrI were annotated in a de novo transcriptome shotgun assembly of reads from tissues of *P. eremicus* (Transcript_74780 and Transcript_129028 in the Pero.BLKT.fasta file at https://doi.org/10.5061/dryad.qf1dp, accessed on 26 July 2023) performed by MacManes and Eisen [28]. We also identified an mRNA from the spleen of *P. boylii*, the brush deermouse, that would encode a FcγRI protein with the expected signal peptide of other mammals (OP524494). 

**Figure 6:**
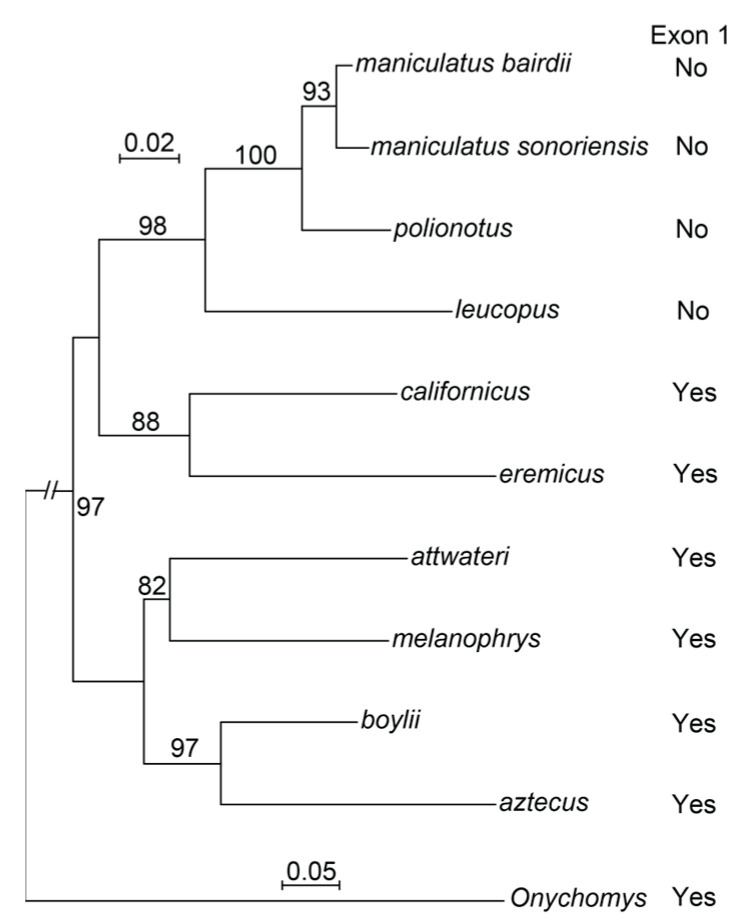
Phylogenetic tree generated by the maximum likelihood method and DNA sequence data from mitochondrial cytochome-*b* genes aligned over 1144 ungapped sites of 9 *Peromyscus* species, including 2 *P. maniculatus* sub-species, and *Onychomys torridus* as the outgroup. The presence or absence of a consensus exon 1 for the Fcgr1 gene in each representative of the species is listed on the right. The % bootstrap nodal support for 1000 iterations is shown. The two size markers are evolutionary distance, with the lower marker of 0.05 applying only to the branch for *O. torridus*. The accession numbers for the sequences are given in the Methods.

Thus, the *Peromyscus* species examined could be divided into two groups: one comprising *P. attwateri*, *P. aztecus*, *P. boylii*, *P. californicus*, *P. eremicus*, and *P. melanophrys*, each of which had the expected coding sequences for the signal peptide of FcγRI, and a second group comprising *P. leucopus*, *P. maniculatus*, and *P. polionotus*, in which the Fcgr1 locus lacked the coding sequences for signal peptides. The two groups mapped to distinct clusters in a phylogram of mitochondrial genomes of these species with an *Onychomys* grasshopper mouse species as the outgroup (Figure 6).

**Figure 7:**
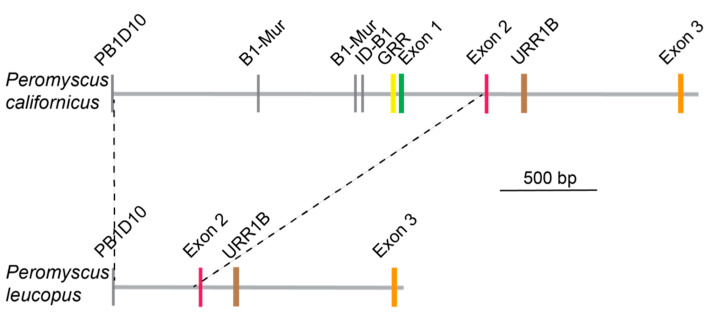
Schematic physical maps of 5′ regions of the Fcgr1 loci in *P. californicus* and *P. leucopus*. SINE retroelements are indicated by gray vertical bars. GRR is the consensus motif for binding of interferon-gamma.

For finer resolution of the differences between two groups, we compared the genome sequences of *P. leucopus* and *P. californicus* in that region (Figure 7). In both species there is a B1 family SINE retrotransposon at the 5′ end, which most closely matched the PB1D10 element of rodents, that was 5′ to the exons. This type of SINE is also present at this location in the *P. maniculatus* reference but not in *O. torridus*. In the *P. californicus* genome, there are SINE repeats of the B1 type (B1-Mur and ID-B1), the consensus binding site for interferon-gamma (GRR), and what corresponds to exon 1 of the *M. musculus* gene. These are not present in either the *P. leucopus* locus (Figure 6) or *P. maniculatus bairdii* (data not shown). In *P. leucopus* there is the predicted exon 2 for Fcgr1, as there is in *P. californicus*. Following this, in both species there is a hAT-Charlie DNA element of the URR1B type and approximately a kilobase further downstream exon 3 of the Fcgr1 gene. 

The genome region between the PB1D10 element and exon 2 in *P. leucopus* and *P. maniculatus* appears to have been deleted, perhaps between tandemly arrayed SINE or other retroelements. What was predicted to be the 5′ end of the protein coding sequence in both *P. leucopus* and *P. maniculatus*, as depicted in Figure 4, is likely an artifact of the NCBI annotation pipeline. The deleted sequence included the coding sequence for the signal peptide of FcγRI and the consensus binding site motif for interferon-gamma in the promoter region [26].

### 3.5. Other Evidence of Pseudogenization

At position 161 of the predicted coding sequence for a FcγRI protein in the reference genome for *P. leucopus* is a translation stop that is not present in deduced proteins in the comparison animals (Figure S1). In the NCBI annotation the in-frame TGA stop codon in the predicted mRNA (XM_037207305) corresponding to chromosome 6 positions 70,726,615–70,726,617 was noted, but for the reference protein sequence the T was substituted to yield the expected open reading frame. Using a targeted PCR for this exon followed by sequencing of the product, we confirmed the genome sequence and the in-frame stop codon in stored DNA of the reference genome animal. With PCR primers bracketing the SNP region, we genotyped 31 other LL stock *P. leucopus*. All 31 colony animals were homozygous for an allele with the in-frame TGA stop codon with a T at the SNP position and designated as the “TT” genotype. 

The genotyping was extended to 32 DNA extracts of blood samples from *P. leucopus* of a wild population in eastern Connecticut [29]. The frequency of the “T” allele was 50% in this population, with a second allele at which a “T” substitutes a CGA arginine codon for the stop (Table 1). The frequencies of the TT, TC, and CC genotypes in the wild Connecticut population were not different than that expected under the Hardy–Weinberg principle (Chi-square test; *p* = 0.65), although the number of individuals examined was modest. If the gene is already inactivated and thus free from evolutionary influences, the substitution of an amino acid for a normally deleterious stop codon could drift to a high frequency. 

## 4. Discussion

Fcgr1 encodes one of a family of cell surface receptors that bind the Fc portion of immunoglobulins [30]. Some receptors, such as FcγRI, are specific for immunoglobulin G, while others bind with the Fc regions of other types of immunoglobulins, for instance IgM in the case of FcµR, encoded by Fcmr. FcγRI differs from the other IgG associated Fc receptors FcγRIIa (CD32), FcγRIIb, and FcγRIII (CD16) in having an affinity for its ligand that is orders of magnitude higher. This greater affinity of FcγRI is conferred by the addition of a third extracellular immunoglobulin-like domain to the two domains found in low-affinity Fc receptors. This allows FcγRI to bind to monomeric IgG as well as immune complexes, while FcγRIIa, FcγRIIb, and FcγRIII are limited to IgG in immune complexes. The proteins that are specific for IgG have the capacity to trigger antibody-dependent cellular cytotoxicity, phagocytosis, and the oxidative burst. FcγRI is constitutively expressed on myeloid cells, which in the blood are mainly granulocytes and monocytes. 

Proteins with homology to Fc receptors for immunoglobulins are found in placental mammals, marsupials, monotremes, birds, reptiles, amphibians, bony fish, and cartilaginous fish but not lower chordates, such as jawless fish or tunicates, or invertebrates [31]. Proteins orthologous to the high-affinity FcγRI appear to be restricted to placental mammals. The percent identity is ≥84% over aligned lengths of ≥68%. Marsupials and monotremes have homologous proteins, but the similarity of these is as great to low-affinity proteins, such as FcγRIII, as to FcγRI, with identities against both being below 45%. Given the ubiquity of Fcgr1 orthologs among mammals, including all families of rodents for which there are genome sequences, the inactivation of this gene in three species of *Peromyscus* but not six others of the genus is notable. *P. leucopus*, *P. maniculatus*, and *P. polionotus* are taxa in a monophyletic clade that is distinct from other *Peromyscus* subgenera (Figure 6) [24]. An estimate of the age of separation of the last common ancestor of *P. leucopus* and *P. maniculatus* from the *Peromyscus* clade containing *P. californicus* is ~3–5 Mya [32].

Between them, *P. leucopus* and *P. maniculatus* are the most abundant mammals in North America after *Homo sapiens* [3,8]. These deermice occupy niches from deserts to mountains. They have adapted well to life among humans and are just as likely to be found in residential areas of suburbs as in pristine woodlands. Given the success of these species, one asks what the consequence of the inactivation of Fcgr1 might have been. Could it be that a few million years ago in North America the spontaneous inactivation of Fcgr1 was of marginal advantage in the most recent common ancestor (MRCA) of these three species? Or perhaps there was a severe bottleneck in the MRCA and an otherwise neutral or even mildly deleterious indel mutation became fixed? 

The full catalog of phenotypes of *M. musculus* with intentionally inactivated Fcgr1 genes remains to be defined. However, reports to date provide some insights regarding the consequences of an inactivated FcγRI gene. Ioan-Facsinay et al. created a knockout by replacing the promoter region, exons 1 and 2, and part of exon 3, with an antibiotic resistance cassette [14]. The location of this inactivation is similar to what we observe in *Peromyscus*. Fcgr1^−/−^ *M. musculus* reportedly developed normally, were fertile, and the main hematopoietic cells in the blood were in their expected frequencies by flow cytometric analysis. As predicted, macrophages of null mutant mice did not bind monomeric IgG, while wildtype macrophages did. In mice treated with LPS the production of IL10, which acts to reverse proinflammatory responses by macrophages, was not affected in Fcgr1^−/−^ mice nor in Fcgr3^−/−^ mice but was attenuated if neither FcγRI nor FcγRIII were expressed; an indication that FcγRIII can perform some of the signaling functions of FcγRI in its absence. In an experimental antigen-induced arthritis model, the Fcgr1^−/−^ mice but not the Fcgr3^−/−^ mice showed decreased cartilage destruction in the joint compared to the wildtype. Moreover, in an experimental model of a *Bordetella pertusis* infection of the respiratory tract, the clearance of the bacteria was impaired in the Fcgr1^−/−^ mice without a discernible difference from wildtype animals in the titers of antibodies to *B. pertussis*.

Barnes et al. constructed a knockout by inserting an antibiotic resistance cassette at the 5′ end of the gene, thereby disrupting the promoter and signal peptide encoding sequence [13]. This yielded FcγRI null macrophages without Fcgr1 sequence transcription, even after treatment with interferon-gamma. The Fcgr1^−/−^ animals were fertile and without developmental abnormalities. The study focused on the binding of ligands to the null mutant cells and effector functions of the macrophages, rather than a broader assessment of the phenotypes of the animals. However, the investigators also demonstrated the following: (i) reduced antibody-dependent cell-mediated cytotoxicity by Fcgr1^−/−^ macrophages using a sheep red blood cell hemolysis assay, (ii) depressed cutaneous inflammation in a reverse passive Arthus reaction model, and (iii) increased IgG responses compared to wildtype after immunization with sheep red blood cells. The latter phenomenon was attributed by the authors to an increase in the number of antibody-forming cells. An overall effect in the null mutant animals showed a tilting toward a Th2-type response instead of a Th1-type response over the time frame of the experiments. 

More recently Zeng et al. used Fcgr1^−/−^ mice resulting from an uncharacterized CRISPR knockout in experiments with a *Chlamydia muridarum* respiratory infection model [33]. The authors reported attenuation of the Th1-type response in the null mutant animals compared to wildtype animals with the infection. Another feature of the mutant phenotype was a shift toward M2-type polarization of lung macrophages rather than the expected M1-type profile. Two manifestations of this macrophage polarization profile were the reduced expression of inducible nitric oxide synthase (Nos2) and the increased expression of interleukin-10 (Il10) during this infection with an obligate intracellular bacterium. 

Brandsma et al. did not disrupt or otherwise inactivate the whole gene but instead assessed the phenotypes of cultured cells transduced with human Fcgr1 alleles representing various nonsynonymous single nucleotide polymorphisms (SNP) that had been observed in low frequencies in human populations [34]. Two missense mutations that are located in the coding sequence for the transmembrane or intracellular domain did not affect the binding of monomeric IgG but did manifest as reduced FcγRI-mediated signaling that was revealed by assays of the MAPK–ERK pathway. Other reported features of a Fcgr1 null mutation in mice are an increased resistance to *Escherichia coli* meningitis that was associated with accelerated clearance of the bacteria from the blood [35], and a reduced antibody-enhanced infectivity of the dengue virus [36]. 

While the experimental systems of these studies were not fully commensurate, what these results indicate is that Fcgr1^−/−^ mice in comparison to wildtype are disposed toward less inflammation in response to either infection or induced hypersensitivity or autoimmunity. This disposition is also a feature of *P. leucopus* exposed to an inflammatory stimulus such as LPS or infection with the relapsing fever agent *Borrelia hermsii* [11,12]. There is insufficient support yet for the claim that the absence of FcγRI function in *P. leucopus* and *P. maniculatus* explains their infection tolerance. For one, it is conceivable that one or another of the low-affinity Fcγ receptors has assumed a high-affinity binding capability after the loss of Fcgr1 in this one clade of *Peromyscus*. On the other hand, prudence on that front need not restrain proceeding with a broader characterization of FcγRI null mutants in *M. musculus*. For example, Fcgr1^−/−^ mice could be compared with wildtype mice for their responses to infection with *B. burgdorferi* or other *Ixodes*-tick-borne pathogens. The macrophages of Fcgr1^−/−^ mice were reported to produce less Tumor Necrosis Factor α in the presence of *B. burgdorferi* but an absence of opsonizing antibody [15], suggesting not only that in early infection *B. burgdorferi* bacteria could more easily evade phagocytosis in null mutant animals but also that they are less likely to elicit inflammation. In regard to this and its other manifestations, this naturally occurring mutation in an abundant and widely distributed rodent may also provide insights about immunopathogenesis of Lyme disease in humans [37]. 

## Figures and Tables

**Table 1:** Fcgr1 allele frequency in a wild population of *Peromyscus leucopus*.

Genotype	Count	Binomial	Expected	Observed
TT	9	p2	0.25	0.28
TC	14	2pq	0.50	0.44
CC	9	q2	0.25	0.28
Total	32		1.00	1.00

## Data Availability

The experiments reported here are associated with National Center for Biotechnology Information (https://ncbi.nlm.nih.gov, accessed on 26 July 2023) BioProjects PRJNA975149 for the combined *P. leucopus* and *M. musculus* experiment and PRJNA996174 for the study of transcriptomes of the spleens of P. *leucopus* infected with *B. burgdorferi* or controls. PRJNA975149 includes 40 BioSamples (SAMN35347136–SAMN35347175) and 40 sets of Sequence Read Archive (SRA) fastq files of Illumina paired-end chemistry reads 1 and 2 (SRR24733648–SRR24733687). BioProject PRJNA996174 includes 11 BioSamples (SAMN36617442–SAMN36617442) and 11 SRA archives of Illumina reads (SRR25385739–SRR253885737).

## Supplementary Materials

The following supporting information can be downloaded at: https://www.mdpi.com/article/10.3390/pathogens12081056/s1. Figure S1: Alignment of partial amino acid sequences of FcγRI proteins of selected rodents, bats, and primates.
