# Supplementary material for: Lyme Disease Agent Reservoirs Peromyscus leucopus and P. maniculatus Have Natively Inactivated Genes for the High-Affinity Immunoglobulin Gamma Fc Receptor I (CD64)
Source: Pathogens. 2023 Aug 18;12(8):1056. doi: 10.3390/pathogens12081056 (PMC10458454; doi:10.3390/pathogens12081056)
Supplement: Supplementary file 1 [file pathogens-2553283-supplementary.pdf › pathogens-2553283-supplementary.pdf]

```
                 31
P leucopus       RAVITLQPPW VSIFQKENVT LWREGPHLPG DSATQWFIHS TLLQISTPRH SITKATFKDS GEYRCRTGLS MPSDPVQLEI HRSWLLLQAS
P maniculatus    RAVITLQPPW VSIFQKENVT LWCEGPHLPG DSATQWFIDS TLLQISTPRH SITKATFKDS GEYGCRTGLS MPSDPVQLEI HRSWLLLQAS
Onychomys        KAVITLQPPW VSIFQKENVT LWCEGLHLPG DSSTQWFINS TLIQISTPRY SITKATFKDS GDYRCRTGLS MPSDPVQLEI HQSWLLLQAS
Mi ochrogaster   KAVITLQPPW VSIFQKENVT LWCEGPHPPG GSSTKWLLNN TVLQVSTPSY RITEATFKDS GEYRCQTGLS MPSDPVQLEI HTELLLLQAS
Mi oregoni       KAVITLQPPW VSIFQEENVT LWCEGPHLPG DSSTQWLLNN TVLQVSTRSY RITEATFKDS GEYRCQTGLS MPSDPVQLEI HTELLLLQAS
Mesocricetus     KAVVTLQPPW VSIFLKENVT LWCEGLHPPG DRTTRWFINS TALQISTPSY SITEATLKDS GEYRCQTGLS MPSDPVQLEI HRDWLLLQAS
Cricetulus       KAVITLQPPW VSIFLKENVT LWCEGLHLPG DSSTQWFINN TLLQVSTPSY SISEATLKDS GEYRCQTGLS MPSDTVQLEI HRDWLLLQAS
Mus musculus     KAVITLQPPW VSIFQKENVT LWCEGPHLPG DSSTQWFING TAVQISTPSY SIPEASFQDS GEYRCQIGSS MPSDPVQLQI HNDWLLLQAS
Mus pahari       KAVITLQPPW VSIFQKENVT MWCEGPHLPG DSSTQWFING TAIQISTPSY SIPEASFQDS GEYRCQRGSS MPSDPVQLQI HSDLLLLQAS
R norvegicus     KAVVTLQPPW LSIFQEENVT LLCEGPHL-G DSSTKWFINS TAIQISTPTY SILKASFKDS GEYKCQTGLS MPSDPVQLEI YRDWLLLQTS
R rattus         KAVVTLQPPW LSIFQEENVT LLCEGPHL-G DSSTKWFINS TAIQISTPTY SILKASFKDS GEYKCQTGLS MPSDPVQLEI YRDWLLLQTS
Rh sinicus       KAVITLQPPW VNIFQMESVT FWCEGPRLPE DNSTQWLVNG TAIRTLTPSY RIASASFSDT GEYRCQTGLS MASDPVQLEI HKDWVLLQVS
Rh ferrumequin.  KAVITLQPPW VNVFQMEKVT LWCEGPRLPG DSSTQWLVNG TVSSVLTHSY SIAAASFSDA GEYRCQTGLS TASDPVQLEI HKDWVLLQVS
Hipposideros     KAVITLQPPW VNVFQMESVT LWCEGPSRPG DSSTQWFLNG TAIHTLAPSY NITAASVKDN GEYTCQTGLS VPSDPVQLEI HKDWLLLQVS
Gorilla          KAVITLQPPW VSVFQEETVT LHCEVLHLPG SSSTQWFLNG TATQTSTPSY RITSASVNDS GEYRCQRGLS GRSDPIQLEI HRGWLLLQVS
Homo sapiens     KAVITLQPPW VSVFQEETVT LHCEVLHLPG SSSTQWFLNG TATQTSTPSY RITSASVNDS GEYRCQRGLS GRSDPIQLEI HRGWLLLQVS

                121                                        *
P leucopus       RRVLTGGEPV TLRCQGWENK LGYNVIFYRN GKSFHF-SXD SEVTVPKSNS SHNGIYHCSG MI--Q-RYTS AGVSVT
P maniculatus    CRILTGGEPL TLRCQGWENK LGYNVIFYRN GKSFHF-SRD SEVTVPESNS SHNGIYHCSG MGRTQ-GYTS AGVSVT
Onychomys        RRVLTEGEPL TLRCHSWQNK LVYKVIFYRN GKSFLF-SRD SEVTILKSTM SHNGIYHCSG MGRSQ-RYTS AGMPVT
Mi ochrogaster   RRVLTEGEPL ALRCHGWKNK LVYNVVFYQN GKSFNF-SRG SEVKILKTNL SHNGVYHCSG MGSNL-RFES AGMAVT
Mi oregoni       HRVLTEGEPL ALRCHGWKNK LVYNVIFYQN GKSFNF-SRG SEVKILKTNL SHNGIYHCSG MGRNL-RFES ARMAVT
Mesocricetus     DRVLTEGKPL TLRCHGWQSK MVYNVVFYRD GKYFHS-SQD SEVTILQTNL THSGVYHCAG IGRHYHRYTS AGMSVT
Cricetulus       HRVLTEGEPL ALRCHGWQSK LVYNVIFYRD GKHFYS-SQD SEVIILQANP SHTGIYHCSG TGRHYRRYTS AGMSVT
Mus musculus     RRVLTEGEPL ALRCHGWKNK LVYNVVFYRN GKSFQF-SSD SEVAILKTNL SHSGIYHCSG TGRH--RYTS AGVSIT
Mus pahari       RRVLTEGEPL ALRCHGWKNK LVYNVVFYRN GKSFQF-SSG SEVVILKTNL NHNGIYHCSG MGRH--RYTS AGVSIT
R norvegicus     HRVLTEGEPL ALRCHPWQNK KVYNVAFYRN GESLDF-SQG SEVTILKTNL SHSGIYHCSA MGKKL--FKS AGVSVT
R rattus         RRVLTEGEPL ALRCHPWQNK KVYNVAFYRN GKSLDF-SQG SEVTILKTNL SHSGIYHCSA MGKNL--FKS AGVSVT
Rh sinicus       SRVVTEGKPL ALRCHGWNNN KLSNMFFYQN RKIFKFSSWD SEFTILRTNM NHNGIYHCSS QIQN--YFIS AGVSIT
Rh ferrumequin.  SRVVTEGKPL ALRCHGWYNN QLSNMLFYQN GKIFKFSSWD PEFTIERTNM SHNDIFHCSG MIRKQ-RFHS AGVSIT
Hipposideros     SRVLTEGKPL ALRCHGWNNM LVYNVVFYQN GKSFKFSSWD SKVTILRTNL SHNGIYHCSS RQKN--HFSS AGISIT
Gorilla          SRVFTEGEPL ALRCHAWKDK LVYNVLYYQN GKAFKFFHWN SNLTILKTNI SHNGTYHCSG MGKH--RYTS AGISVT
Homo sapiens     SRVFTEGEPL ALRCHAWKDK LVYNVLYYRN GKAFKFFHWN SNLTILKTNI SHNGTYHCSG MGKH--RYTS AGISVT
```
